# SPECT imaging of pulmonary vascular disease in bleomycin-induced lung fibrosis using a vascular endothelium tracer

**DOI:** 10.1186/s12931-021-01836-3

**Published:** 2021-09-04

**Authors:** François Harel, Quang T. Nguyen, Mohamed J. Nsaibia, Vincent Finnerty, Arielle Morgan, Martin Sirois, Louis Villeneuve, Angelino Calderone, Alexandre Bergeron, Emmanuelle Brochiero, Jean-Claude Tardif, YanFen Shi, Jocelyn Dupuis

**Affiliations:** 1 Montreal Heart Institute Research Center, 5000 Belanger Street, Montreal, QC H1T 1C8 Canada; 2 Department of Radiology, Radio-Oncology and Nuclear Medicine, Faculty of Medicine, Université de Montréal, 2900 Edouard-Montpetit Boulevard, Montreal, QC H3T 1J4 Canada; 3 Department of Pharmacology and Physiology, Faculty of Medicine, Université de Montréal, 2900 Edouard-Montpetit Boulevard, Montreal, QC H3T 1J4 Canada; 4 Department of Medicine, Faculty of Medicine, Université de Montréal, 2900 Edouard-Montpetit Boulevard, Montreal, QC H3T 1J4 Canada; 5 Centre de Recherche du Centre Hospitalier de l’Université de Montréal (CRCHUM), 900 Saint-Denis Street, Montreal, QC H2X 0A9 Canada

**Keywords:** Molecular imaging, Adrenomedullin, Pulmonary hypertension, Pulmonary embolism, Nuclear medicine

## Abstract

**Background:**

Pulmonary hypertension (PH) complicating idiopathic pulmonary fibrosis (IPF) is associated to worse outcome. There is a great need for a non-invasive diagnostic modality to detect and evaluate the severity of pulmonary vascular disease (PVD). ^99m^Tc-PulmoBind is a novel imaging agent that binds to the adrenomedullin (AM) receptor on the pulmonary microvascular endothelium. SPECT imaging employing the endothelial cell tracer ^99m^Tc-PulmoBind was used to assess PVD associated with lung fibrosis.

**Methods:**

Rats with selective right lung bleomycin-induced fibrosis were compared to control rats. SPECT imaging was performed after three weeks with ^99m^Tc-PulmoBind and ^99m^Tc-macroaggregates of albumin (MAA). PH and right ventricular (RV) function were assessed by echocardiography. Lung perfusion was evaluated by fluorescent microangiography. Lung AM receptor expression was measured by qPCR and by immunohistology. Relevance to human IPF was explored by measuring AM receptor expression in lung biopsies from IPF patients and healthy controls.

**Results:**

The bleomycin group developed preferential right lung fibrosis with remodeling and reduced perfusion as assessed with fluorescent microangiography. These rats developed PH with RV hypertrophy and dysfunction. ^99m^Tc-PulmoBind uptake was selectively reduced by 50% in the right lung and associated with reduced AM receptor expression, PH and RV hypertrophy. AM receptor was co-expressed with the endothelial cell protein CD31 in alveolar capillaries, and markedly reduced after bleomycin. Quantitative dynamic analysis of ^99m^Tc-PulmoBind uptake in comparison to ^99m^Tc-MAA revealed that the latter distributed only according to flow, with about 60% increased left lung uptake while left lung uptake of ^99m^Tc-PulmoBind was not affected. Lung from human IPF patients showed important reduction in AM receptor expression closely associated with CD31.

**Conclusions:**

SPECT imaging with ^99m^Tc-PulmoBind detects PVD and its severity in bleomycin-induced lung fibrosis. Reduced AM receptor expression in human IPF supports further clinical development of this imaging approach.

**Supplementary Information:**

The online version contains supplementary material available at 10.1186/s12931-021-01836-3.

## Background

Chronic lung diseases, including idiopathic pulmonary fibrosis (IPF), can be associated with pulmonary vascular disease (PVD) [1] leading to pulmonary hypertension (PH). Reports suggest a PH prevalence of 8%-15% in the early stages to 30%-50% in advanced IPF [2]. The recently revised definition of PH lowered the diagnostic threshold to a mean PAP > 20 mmHg and therefore likely increases the prevalence [3].

Subjects with IPF and PH have worse functional and vital prognosis [2, 4]. Specific PH therapies have not shown benefit and are not recommended for IPF. A recent review concluded that in view of the high prevalence of chronic lung diseases and the much worse prognosis conferred by associated PH: “further investigations are required in order to improve early diagnosis and provide better clinical management” [4]. Indeed, there is no imaging modality that can provide early specific diagnosis of PVD.

PulmoBind is a molecular SPECT imaging agent developed for the diagnosis and evaluation of PVD [5–9]. Radiolabeled with ^99m^Tc, it binds with high affinity to the adrenomedullin (AM) receptor densely expressed by the vascular endothelium in alveolar capillaries. PulmoBind detects PVD in animal models of group I PH [5, 8]. Phase I and II human trials were also completed and PulmoBind successfully detected PVD in patients with pulmonary arterial hypertension (group I PH) and with chronic thromboembolic PH (group IV PH) [7, 10]. We hypothesized that PulmoBind could be useful for the detection of PVD in chronic lung disease (group III PH) and designed experiments in the established model of IPF induced by bleomycin [11]. To test the capacity to detect heterogeneous distribution of PVD, we used an asymmetrical model causing preferential right lung injury and compared PulmoBind distribution to that of fluorescent microspheres. We also investigated the added value of molecular imaging with PulmoBind by quantitative comparison of lung perfusion imaging using ^99m^Tc-labelled macroaggregates of albumin (MAA), a physical tracer that distributes exclusively according to flow. Furthermore, we determined the expression of the AM receptor in relation to PulmoBind lung uptake. Finally, the potential clinical relevance was evaluated by comparing lung expression of the AM receptors in controls and IPF patients.

## Methods

### Model of selective right lung injury and fibrosis induced by bleomycin

In order to test the capacity of PulmoBind to detect localized PVD in lung fibrosis we used a model of selective right lung fibrosis. Male Wistar rats (175–200 g, Charles River) were anesthetized using 3% isoflurane in 100% of oxygen. A single intratracheal instillation of 0.3 mL bleomycin (3 mg/kg) or 0.9% saline solution was achieved with rats at a 45-degree right-lateral decubitus and 45-degree cranio-caudal angulation so that the right hemithorax was lower. Following instillation, the animals rested on the right side for 2 min. The animals’ experimental measurements were performed 3 weeks later.

### Echocardiographic study

In order to evaluate the impact of PVD on right ventricular hypertrophy and function we performed cardiac ultrasound studies. Rats were sedated by 1.5–2% isoflurane. M-mode was used to measure right ventricular (RV) dimension and RV anterior wall thickness at end-diastole (RVDd, RVAWd). RV function was estimated by measuring the tricuspid annulus plane systolic excursion (TAPSE). Pulmonary artery acceleration time (PAAT) was measured by pulmonary artery flow recorded by pulsed wave Doppler. This parameter, indicative of increased RV afterload is inversely associated to the severity of PH. The average of three consecutive cardiac cycles was used for each measurement.

### In vivo lung uptake of ^99m^Tc-MAA and ^99m^Tc-PulmoBind

In order to determine whether lung uptake of ^99m^Tc-PulmoBind, a molecular tracer, differed from that of ^99m^Tc-MAA, a physical tracer that distributes according to flow, we performed comparative experiments in the asymmetrical model of bleomycin lung fibrosis. Animals were anesthetized by isoflurane (1.5–2%). ^99m^Tc-MAA (Draximage) was prepared and particles size verified to be between 10 and 50 μm. 300 μL of ^99m^Tc-MAA (14.4 ± 8.2 MBq) was injected via the caudal vein. Whole-body biodistribution was measured by a nuclear medicine camera (Ecam, Siemens) equipped with a low-energy high resolution parallel-hole collimator. Dynamic acquisitions were recorded for a 30-min period, and static whole-body scan was obtained at 30 min. To produce higher-resolution tomographic images, HiSPECT APT-5 collimators (Multiplexed multi-pinhole SPECT colimators from SciVis, Gottingen Germany) were used. Three days later, the same animal had repeated imaging with ^99m^Tc-PulmoBind (24.7 ± 5.0 Mbq) prepared as previously described [7]. PulmoBind drug substance (American Peptide Company, CA, USA) and diagnostic kits (KABS, St-Hubert, QC, Canada) were produced according to good manufacturing practices with drug substance purity > 98%. Each labeling kit contained 18.5 μg (4.33 nmol) of drug substance. Radiochemical purity was verified by instant thin layer chromatography.

Dynamic image acquisitions were evaluated with MATLAB version 7.01. Tomographic images were reconstructed with an OSEM algorithm on the dedicated software HiSPECT 1.4.2611 (2011 SciVis, Gottingen Germany). Results were expressed as the percentage of the injected dose.

### Fluorescent microangiography

In order to visualize lung vasculature and detect PVD with great resolution in the asymmetrical model of bleomycin lung fibrosis, we used fluorescent microangiography. The technique relies on low-temperature melting point agarose with fluorescent polystyrene microspheres [12]. In brief, rats were anaesthetized, ventilated and the pulmonary vessels flushed with heparinized saline. Three mL of prewarmed solution containing 10% of 0.2 µm diameter of fluorescent microbeads (FluoSpheres; Invitrogen) reconstituted in low-temperature melting point agarose was infused at controlled pressure of 25 or 60 mmHg for sham and bleomycin respectively. The chest was covered with crushed ice to enhance gelling of the perfusate. The lung was then instilled intratracheally with cold buffered 10% formalin. Lung blocs were embedded with optimum cutting temperature compound and stored at − 80 °C overnight. 50-μm-thick cross sections of the lungs were analyzed by fluorescent microscopy by collecting a z-series through the sections.

### Assessment of lung, heart and body weights

After the last imaging procedure, animals were weighed and administered a lethal dose of Ketamine/xylazine followed by exsanguination. The heart (right and the left ventricle plus septum) and the lungs were excised for weight, snap-frozen and stored at − 80 °C for molecular biology.

### Histological examination

Histological experiments were performed to determine the expression of the adrenomedullin receptor and its relation to the vascular endothelium by also evaluating the expression of the platelet endothelial cell adhesion molecule (CD31). Expression was evaluated in both controls and bleomycin lung fibrosis rats and their tissue localization was assessed by using confocal microscopy. Lung tissue samples were fixed in 10% neutral buffered formalin for histological examination using Masson’s trichrome staining. Immunofluorescence studies were performed by using tissue sections that were deparaffinized, hydrated and exposed to the antigen retrieval method (citrate-based solution pH 6.0) at a temperature of 100 °C for 30 min. Primary antibodies used include mouse monoclonal anti-RAMP2 (1:40; Santa Cruz Biotechnology, Santa Cruz, CA), rabbit polyclonal anti-CD31 (1:200; Novus Biologicals, Littleton, CO). The nucleus was identified with 4′,6′-diamidino- 2 phenylindole (DAPI, 1.5 μM; emission wavelength, 460 nm; Sigma-Aldrich) staining. Secondary antibodies used include donkey anti-rabbit IgG conjugated to Alexa-555 (1:600; Invitrogen, Carlsbad, CA, USA), donkey anti-mouse IgG conjugated to Alexa-647 (1:600; Invitrogen, Carlsbad, CA, USA). Non-specific staining was determined following the addition of the conjugated secondary antibody alone. Confocal imaging was performed with the use of either a 10X or a 63X oil-immersion, 1.4 numerical aperture DIC plan apochromat objective lens mounted on a Zeiss Axiovert 100 M confocal microscope.

### Adrenomedullin receptor expression in rat lung

These experiments were performed to determine whether lung uptake of PulmoBind, a specific adrenomedullin receptor ligand, was associated with reduce expression of this receptor in the lungs of rats with bleomycin-induced fibrosis. RNA was extracted using the Qiagen TRIzol reagent (Fisher Scientific) and treated with TURBO DNA-free DNase (Fisher Scientific). Extracted RNA was converted to cDNA using GoScript Reverse Transcriptase with 500–1000 ng starting material. The quality of total RNA was monitored by capillary electrophoresis (Experion, Biorad, ON, Canada). Quantitative real-time PCR (qPCR) was performed on an AB-7900HT real-time cycler using TaqMan gene expression assays (Life Technologies). Primers for RAMP2 and CLR (rat) were obtained from Life Technologies. qPCR data was analyzed using the ΔΔCt method, using Ubiquitin C (UBC) or GAPDH as normalization controls for lung tissue.

### Adrenomedullin receptor expression in human lung

To explore the potential clinical relevance of our findings in a rat model of lung fibrosis and the potential utility of PulmoBind in the imaging of PVD in human lung fibrosis, we determined adrenomedullin receptor expression in human lungs with and without idiopathic pulmonary fibrosis. Protein lysates of lung tissues were prepared and subjected to SDS-electrophoresis. Antibodies used include the mouse monoclonal anti-vimentin (1:500); the mouse monoclonal anti-RAMP2 (1:400); the mouse monoclonal anti-SM22α (1:50.000), and the mouse monoclonal anti-αSMA (1:50.000) all from Santa Cruz Biotechnology, Santa Cruz, CA. The rabbit polyclonal anti-CD31 (1:1000; Novus Biologicals, Littleton, CO) and a mouse monoclonal anti-GAPDH (1:50,000; Ambion, Austin TX). Detection was done using clarity western ECL substrate (Perkin Elmer, Waltham, MA). Images were acquired, and quantification analyses were performed using a ChemiDocMP system (BioRad, ON, Canada) and densitometric analyses of Western blot were performed using ImageJ software. Protein signal was normalized to GAPDH.

### Statistical analyses

Differences between groups were evaluated by two-tailed Mann–Whitney tests with a level of significance at p < 0.05. Relationship between individual parameters was evaluated by simple linear regression analysis. All results are reported as mean ± SEM and all statistical analysis were performed using Prism software (version 8.4.3; GraphPad).

## Results

**Fig. 1:**
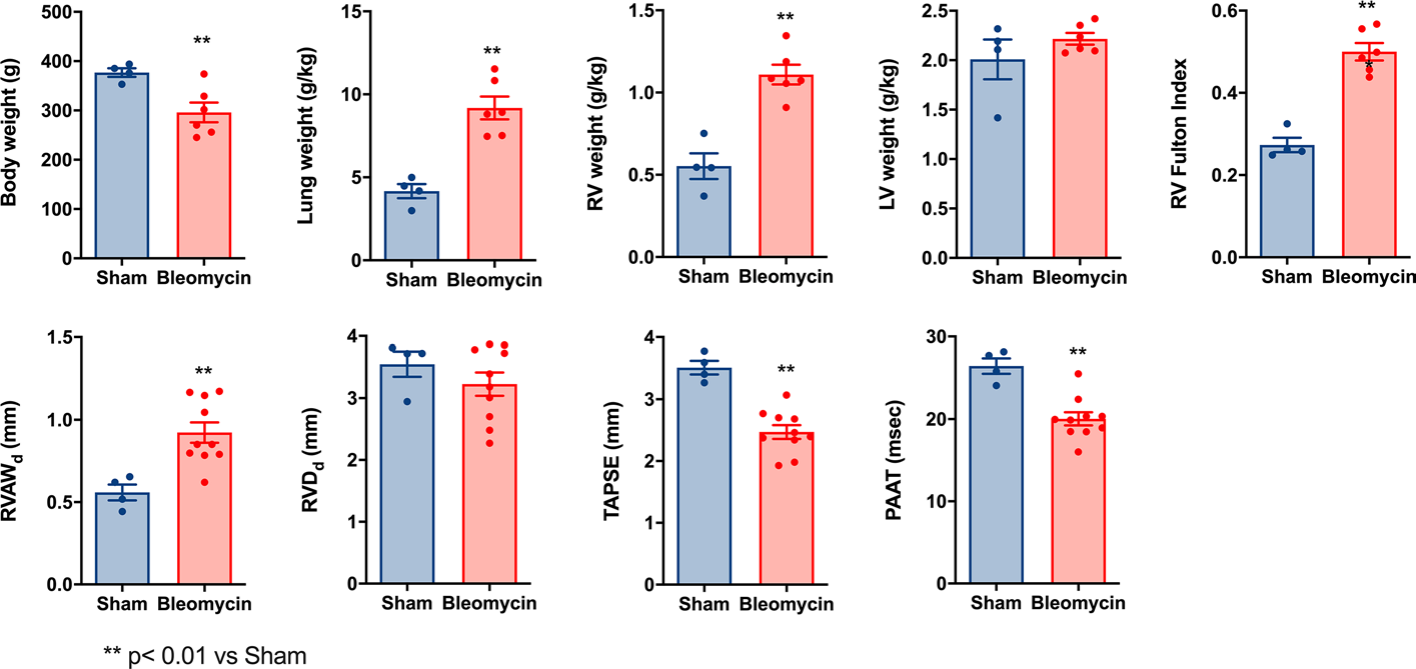
PH and lung remodeling after bleomycin. Effect of bleomycin-induced lung fibrosis on body, lung and right ventricular weights (upper graphs, sham n = 4; Bleomycin n = 6), and on cardiac ultrasound parameters (lower graphs, sham n = 4; Bleomycin n = 10). *LV* left ventricle, *PAAT* pulmonary artery acceleration time, *RV* right ventricle, *RVAW* right ventricle anterior wall thickness, *RVD* right ventricle dimension, *TAPSE* tricuspid annulus plane systolic excursion. **p < 0.01 vs Sham

Three weeks after administration, body weight was reduced in bleomycin-treated rats whereas lung and RV weights increased twofold (Fig. 1). There was no difference in left ventricle weight and consequently, severe RV hypertrophy measured from the Fulton index (Fig. 1). The latter data was confirmed at echocardiography with almost doubling of RV wall thickness. The RV was not dilated but its function, evaluated by TAPSE, was reduced. There was evidence of PH with increased RV afterload translating to a reduction in PAAT (Fig. 1). These data demonstrate that bleomycin caused important lung remodeling leading to PH, RV hypertrophy and RV dysfunction.

### Selective right lung injury and fibrosis cause reduced right lung perfusion (Fig. 2)

**Fig. 2:**
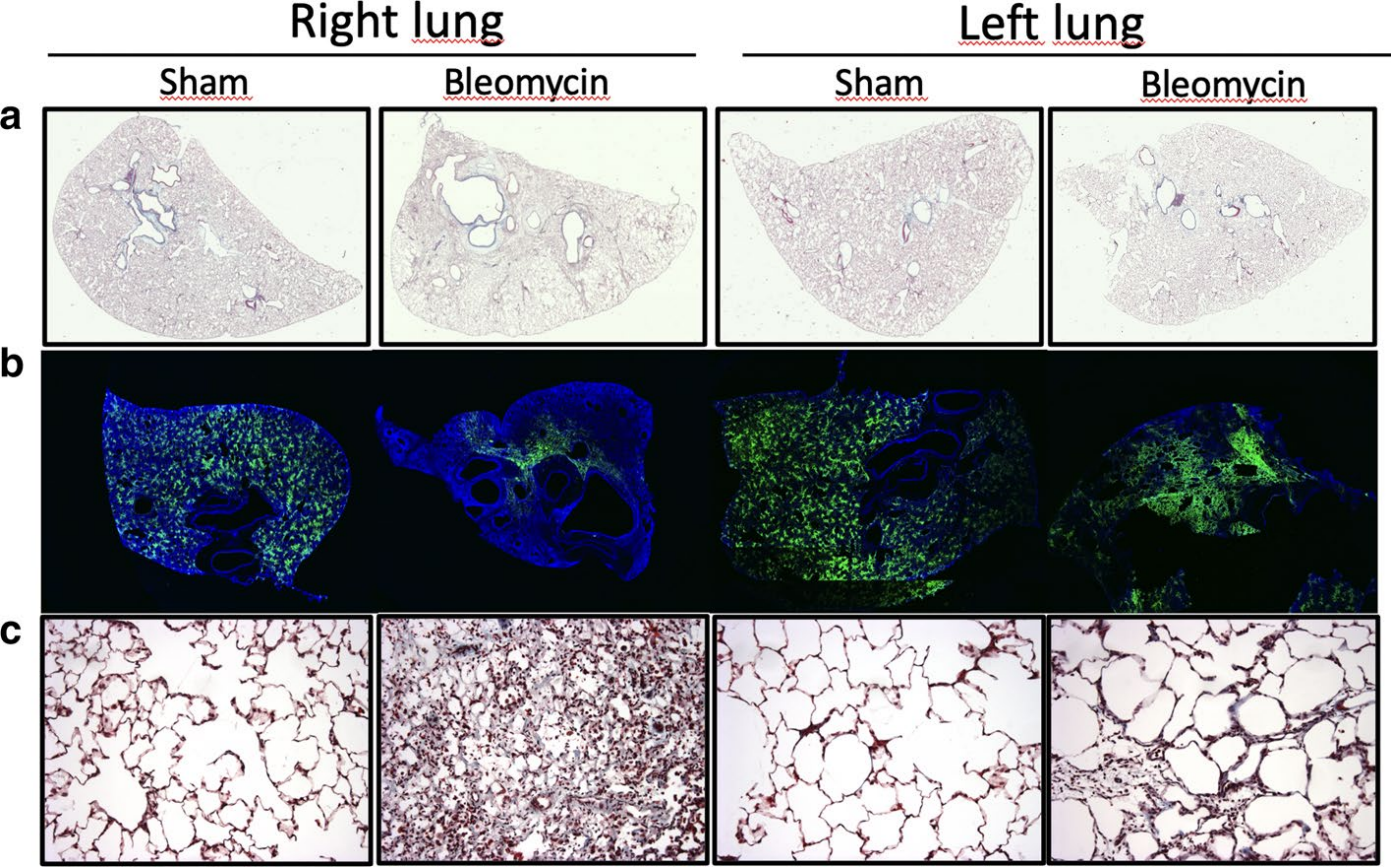
Preferential right lung fibrosis and reduced perfusion. **a** Histological section of the whole right and left lungs from sham and bleomycin animals. **b** Fluorescent microangiography of sections from whole right and left lung. **c** Massons’s trichrome staining of lung section at 40X showing fibrosis (in blue), increased cellularity and alveolar wall thickness in the right lung after bleomycin injury

Postural positioning of rats resulted in preferential right lung injury and fibrosis evidenced at histological examination (Fig. 2). At higher magnification (Fig. 2c) there is important fibrosis and alveolar thickening in the right lung with less severe injury in the left. Lung tissue perfusion assessed by fluorescent microangiography shows homogeneous flow distribution in both the right and left lung of sham animals. However, an important reduction of perfusion after bleomycin injury was more prominent in the right versus the left lung (Fig. 2b).

### Non-invasive imaging of asymmetrical lung perfusion with ^99m^Tc-PulmoBind (Fig. 3)

**Fig. 3:**
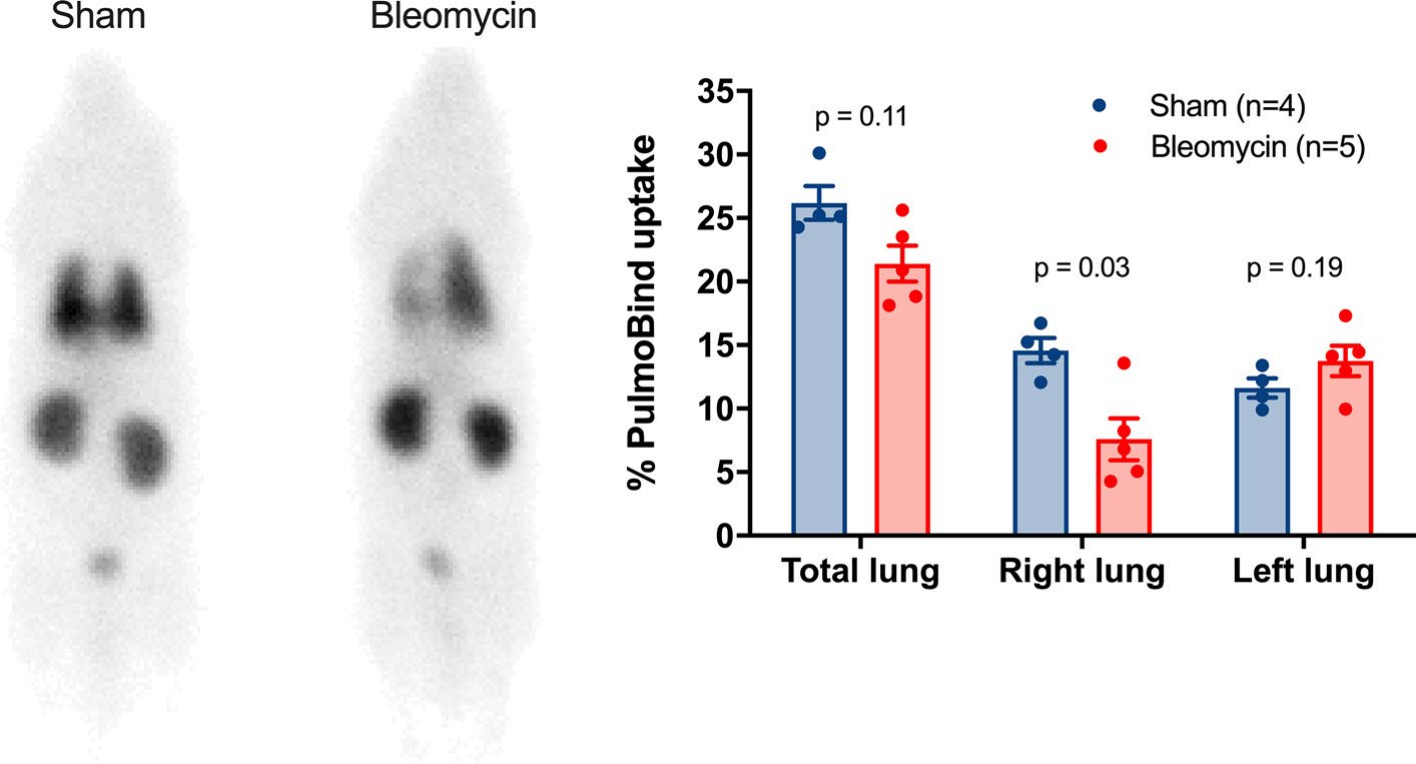
Reduced right lung uptake of ^99m^Tc-PulmoBind. Whole body planar imaging of a rat with ^99m^Tc-PulmoBind 10 min after injection in a sham and a bleomycin treated animal (left) and bar graph of the mean uptake after 10 min in sham (n = 4) and in bleomycin treated animals (n = 5)

Planar imaging 10 min after ^99m^Tc-PulmoBind injection shows homogeneous uptake in the lungs and to a lesser extent in the kidneys. In bleomycin-treated rats, an important reduction of uptake was observed in the right lung. These data were confirmed by quantification showing that mean % lung uptake was non-significantly reduced for the total lung, but significantly reduced by ~ 50% in the right lung with no significant difference in the left lung.

### Lung uptake of ^99m^Tc-PulmoBind compared to ^99m^Tc-MAA (Figs. 4, 5)

**Fig. 4:**
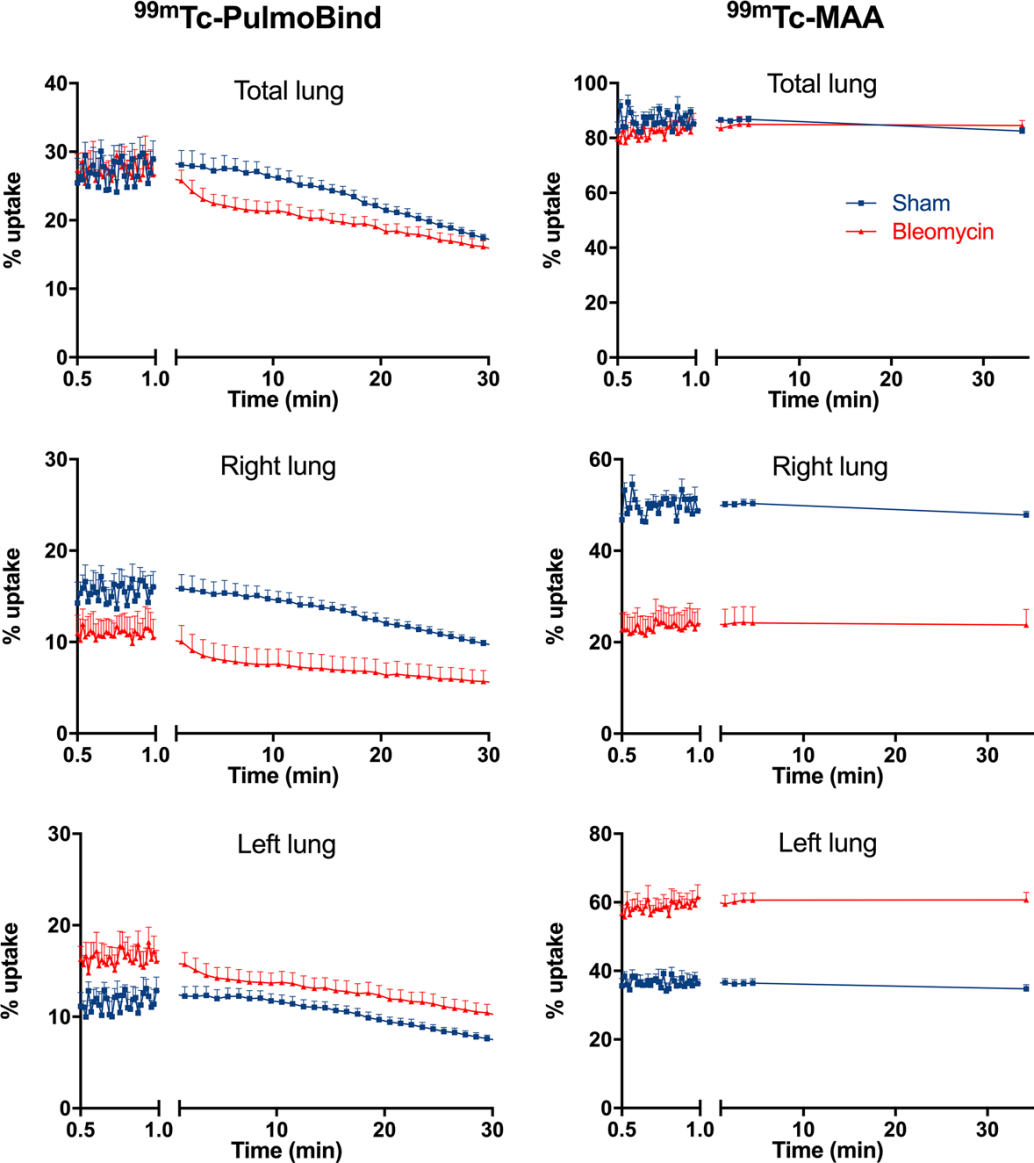
Comparison of ^99m^Tc-PulmoBind and ^99m^Tc-MAA. Dynamic uptake-time curves for ^99m^Tc-PulmoBind and ^99m^Tc-MAA in total, right and left lungs from sham (n = 4) and bleomycin-treated animals (n = 5). *MAA* macroaggregates of albumin, *Tc* technetium

**Fig. 5:**
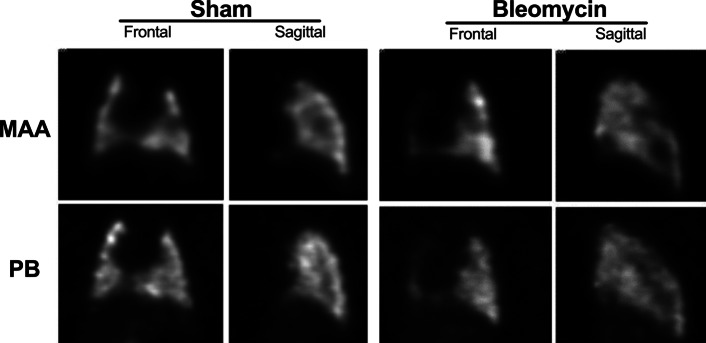
SPECT imaging with ^99m^Tc-PulmoBind and ^99m^Tc-MAA. SPECT images in the frontal and sagittal planes for ^99m^Tc-PulmoBind (PB) and ^99m^Tc-MAA (*MAA* macroaggregates of albumin) from sham and bleomycin-treated animals

Dynamic lung uptake versus time curves are shown (Fig. 4) after injection, with analysis for the total lung, and for the right and left lung separately. Total lung uptake of ^99m^Tc-PulmoBind was slightly lower in the bleomycin group while the injected ^99m^Tc-MAA was maximally retained by the total lung in both sham and bleomycin groups with no difference and perfectly superposable curves. In the right lung, ^99m^Tc-PulmoBind was markedly reduced by about 50% after bleomycin, and ^99m^Tc-MAA uptake was even more markedly reduced by about 60%. The different behavior of the tracers in the left lung was even more striking: ^99m^Tc-PulmoBind uptake was quite similar to the sham treated animals, but ^99m^Tc-MAA uptake was much higher in the left lung of the bleomycin group with an approximately 50% increase.

SPECT images in the frontal and sagittal views are shown in Fig. 5 for both PulmoBind and MAA in sham and bleomycin treated animals. Both agents resulted in similar normal lung pictures in the sham animals. In the bleomycin treated group, there was marked reduction of uptake in the right lung that seems similar with both ^99m^Tc-PulmoBind and ^99m^Tc-MAA as a tracer. Upon close inspection however, and concordant with data from Fig. 4, there is more visible right lung uptake of ^99m^Tc-PulmoBind in the bleomycin animal while left lung uptake of ^99m^Tc-MAA seems higher.

### Effect of bleomycin-induced lung fibrosis on AM receptor expression (Figs. 6, 7a)

**Fig. 6:**
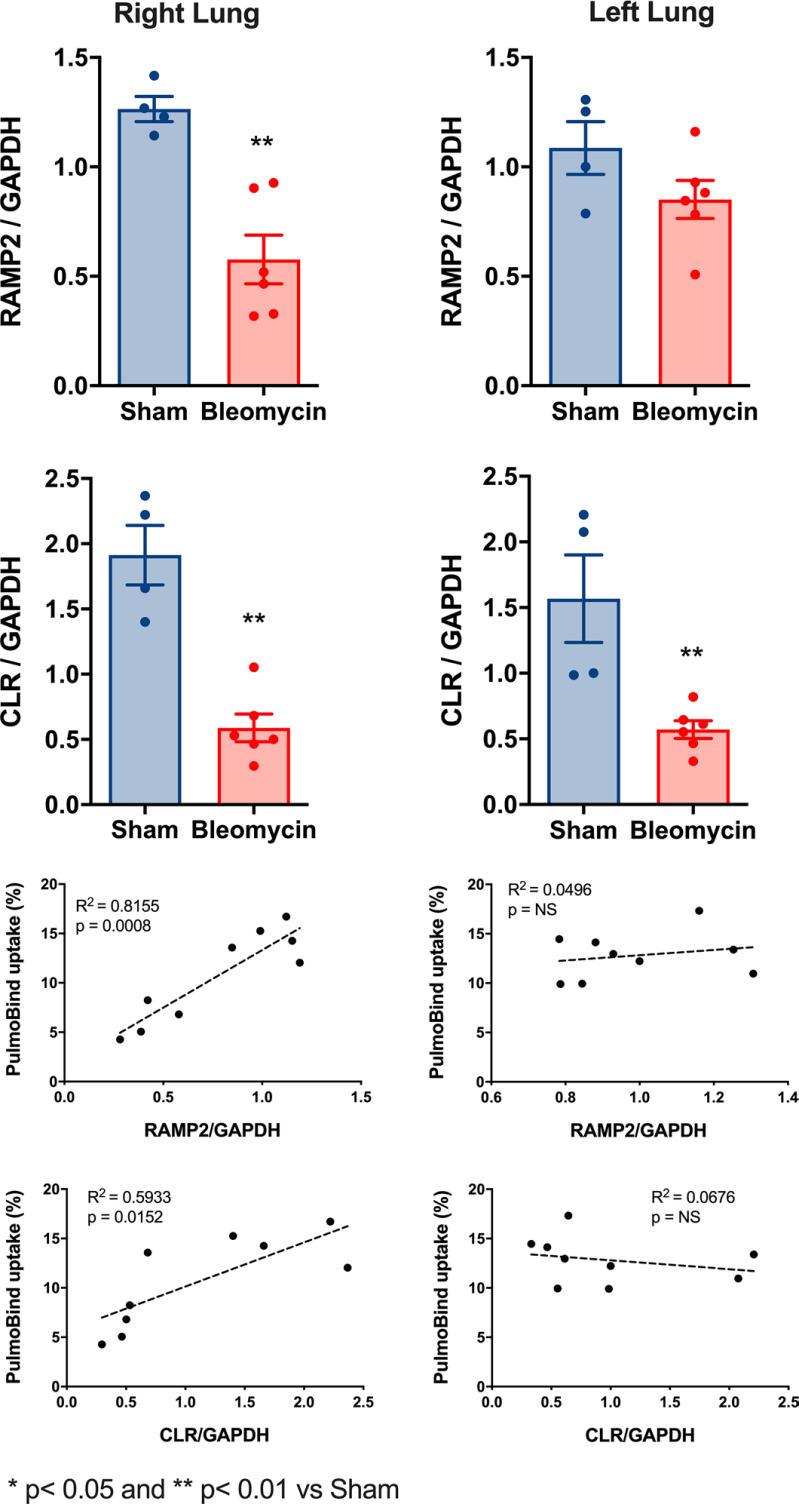
Reduced adrenomedullin (AM) receptor expression in the right lung. Expression of RAMP2 and CLR components of the AM receptor by qPCR and simple linear regression analysis in the right and left lung from sham and bleomycin-treated animals. Sham (n = 4) and bleomycin-treated (n = 6). ^99m^Tc-PulmoBind uptake not performed in one bleomycin animal for technical reasons. *CLR* calcitonin like receptor, *RAMP2* receptor activity modifying protein 2. *p < 0.05 and **p < 0.01 vs Sham

**Fig. 7:**
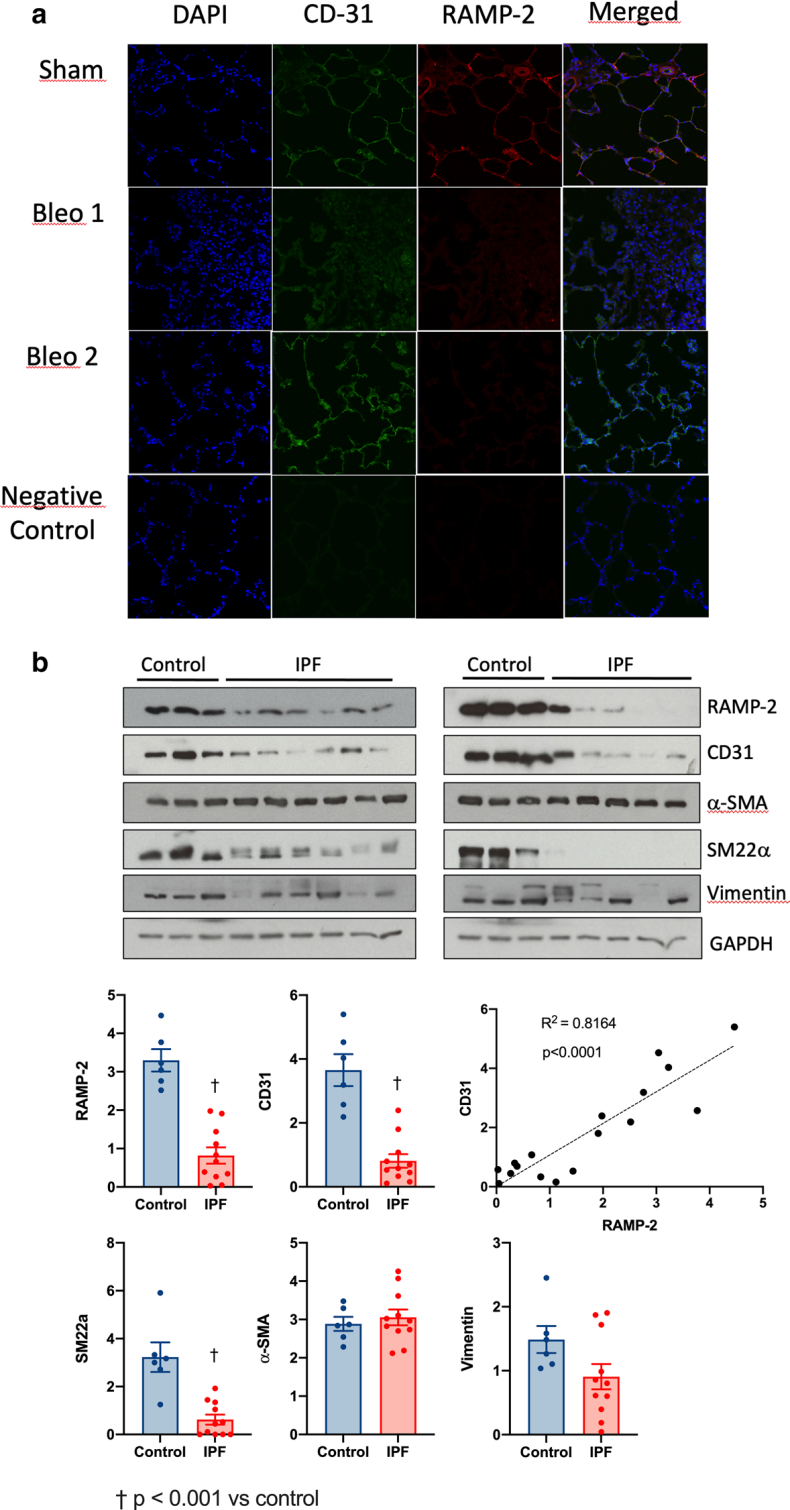
**a** Reduced AM receptor expression in lung vasculature. Confocal imaging of lung sections from a sham and two bleomycin-treated animals (bleo1 and bleo2) compared to a negative control. *RAMP2* receptor activity modifying protein 2. **b** Reduced expression of AM receptor in humans with IPF. Lung tissue protein expression of the AM receptor (RAMP2) and of other molecular markers in lungs from humans with IPF (n = 11) and control lungs (n = 6). †P < 0.001 vs control. *αSMA* alpha-smooth muscle actin, *IPF* idiopathic pulmonary fibrosis, *RAMP2* receptor activity modifying protein 2, *SM22α* smooth muscle protein 22-alpha

The specific AM receptor is a heterodimer composed of the receptor activity modifying protein 2 (RAMP2) and the calcitonin-like receptor (CLR). In the bleomycin group, RAMP2 expression was significantly reduced in the right, but not in the left lung (Fig. 6). CLR expression was significantly reduced in both the right and the left lung. Lung uptake of ^99m^Tc-PulmoBind was associated with both RAMP2 and CLR expressions in the right, but not in the left lung (Fig. 6). Furthermore, reduced AM receptor expression in the right lung was also associated with the severity of PH (PAAT) and with reduced RV function (TAPSE) measured at echocardiography (Additional file 1: Fig. S1).

The effect of bleomycin on pulmonary vascular endothelium integrity and AM receptor expression was evaluated by immunohistology with confocal imaging (Fig. 7a). Two bleomycin-treated rats and one negative control are shown compared to a sham animal. In the sham animal, the endothelial cell tracer CD31 shows distribution in the alveolar walls with co-expression of RAMP2. The bleomycin treated animals showed increased lung cellularity evidenced by greater DAPI staining. In the first bleomycin-treated rat (Bleo1), expression of CD31 and of RAMP2 was absent, confirming the absence of functional capillaries. In the second bleomycin-treated rat (Bleo 2), CD31 expression wat detected whereas RAMP2 was absent, suggesting the presence of capillaries lacking RAMP2 expression. The negative control confirms the specificity of the CD31 and RAMP2 antibodies.

### Expression of an endothelial cell tracer and of the AM receptor in humans with IPF (Fig. 7b)

To explore the potential relevance of using PulmoBind in human IPF, we measured the expression of CD31 and of the selective AM receptor component (RAMP2) in lungs from 3 controls and 6 IPF patients. IPF patients showed marked reductions of both CD31 and RAMP2 expression. There was a close linear relationship between the two suggesting co-expression in the pulmonary vasculature. Smooth muscle protein 22-alpha (SM22a), a human smooth muscle specific protein, was also reduced suggesting a reduction in lung vessels. Alpha-smooth muscle actin (α-SMA), a protein also expressed by lung myofibroblasts, was however not reduced in the IPF subjects, as was the intermediate filament protein vimentin.

## Discussion

There is great need for a non-invasive imaging modality able to specifically detect the presence and determine the severity of PVD associated with chronic lung disorders such as IPF. For that purpose, we evaluated the vascular endothelium tracer ^99m^Tc-PulmoBind using qualitative and quantitative SPECT imaging in bleomycin-induced lung fibrosis.

### Preferential right lung injury results in more severe PVD

Selective right lung instillation of bleomycin caused greater right lung injury and fibrosis. This was accompanied by a greater degree of PVD with marked reduction of right lung perfusion by fluorescent microangiography. Since PulmoBind is a specific high affinity synthetic ligand of the AM receptor expressed by the vascular endothelium [8], the marked reduction of uptake by the right lung confirms reduced lung tissue perfusion due to vascular disease.

AM is part of the calcitonin gene-related peptide family and binds to a heterodimeric receptor composed of RAMP2 and CLR, RAMP2 conferring AM specificity [13]. Studies have shown that the AM-RAMP2 system is a key determinant of vascular integrity and homeostasis [14]. We found that expression of both components of the receptor were significantly reduced in the right lung of bleomycin exposed animals. Furthermore, immunohistology studies showed that RAMP2 is principally expressed in alveolar capillaries with co-localization with the platelet/endothelial cell adhesion molecule (CD31), an endothelial cell surface protein. Confocal imaging further showed that RAMP2 and CD31 expressions were significantly reduced in the right lung with some areas displaying complete absence of staining of both proteins (bleo1, Fig. 7) while other areas showed maintained expression of CD31, but without RAMP2 expression (bleo2, Fig. 7). Furthermore, ^99m^Tc-PulmoBind uptake was associated by simple regression analysis with RAMP2 expression in the right lung as well as with the severity of RV dysfunction (TAPSE) and of PH (PAAT). Altogether, these data demonstrate that lung ^99m^Tc-PulmoBind uptake, a reflection of endothelial AM receptor functional integrity, can be used to evaluate the severity of PVD in this model.

### Comparison of ^99m^Tc-PulmoBind and ^99m^Tc-MAA uptake kinetics

There are important differences between ^99m^Tc-PulmoBind, a vascular endothelium molecular tracer, and ^99m^Tc-MAA, a physical tracer that distributes solely according to flow. ^99m^Tc-PulmoBind uptake reveals tracer-accessible endothelial vascular surface expressing functional AM receptors [8]. Single-pass indicator-dilution experiments with labelled AM in dogs showed that AM uptake occurs according to a single rate constant without return of tracer into circulation within a single transit time, compatible with a single receptor high affinity binding [15]. Another important inherent property of ^99m^Tc-PulmoBind resides in its small molecular dimension coupled with the dense alveolar capillary distribution of its receptors [16, 17]: binding of the tracer occurs in the microcirculation. ^99m^Tc-PulmoBind uptake by the lungs therefore depends on flow to regions of the lungs that maintain expression and function of the adrenomedullin receptor. ^99m^Tc-MAA, on the other hand, is a purely physical tracer composed of aggregates selected to be of 10 to 50 µm with injection of up to 500,000 aggregates per test in humans.^99m^Tc-MAA is a large tracer that distributes according to flow, the injected aggregates “plugging” as many vessels of inferior diameter.

In the current study where we looked at uptake after selective right lung fibrosis causing reduction of right lung perfusion proven by fluorescent microangiography, we found markedly reduced right lung flow with ^99m^Tc-MAA and also, but to a lesser extent, reduced PulmoBind uptake. In the left lung where there is less fibrosis and preserved vasculature as assessed by fluorescent microgangiography, we have found markedly increased flow as evidenced by increased ^99m^Tc-MAA uptake, but no significant change in PulmoBind uptake. This confirms that PulmoBind uptake mirrors the loss of vasculature (PVD) in the right lung and the preserved vasculature in the left lung. The behavior of ^99m^Tc-PulmoBind confirms the greater sensitivity of an endothelial cell specific tracer to detect the presence and the extent of PVD. The results further support that combination of the two tracers together provides useful information on PVD by combining the knowledge of flow distribution with ^99m^Tc-MAA, and that of the functional microcirculation as assessed by ^99m^Tc-PulmoBind. This suggests that dual modality imaging evaluating pulmonary blood flow and pulmonary endothelial function may indeed provide better phenotyping of pulmonary vascular disease.

### Potential relevance of these findings in humans with IPF

Previous investigators have established that the AM receptor is densely expressed in normal human alveolar capillaries [17] and that IPF patients demonstrate reduced vascular density evidenced by reduced CD31 expression in fibroblastic foci [1]. In lung biopsies from human subjects with IPF, AM receptor and CD31 protein were markedly reduced and a linear relationship was observed between the two proteins. This suggests that PVD and loss of endothelium in these subjects is associated with reduced AM receptor expression. These findings certainly support further investigation of ^99m^Tc-PulmoBind as a marker of PVD in IPF. The mechanisms of pulmonary vascular disease in IPF are still unclear and molecular imaging using an endothelium tracer at different stages of the disease would help expand comprehension of this area of research and help development of therapeutics.

### Limitations of this study

The findings of this study in an animal model of lung fibrosis cannot be extrapolated to human IPF. The small sample size for some experiments also represents a weakness of this study. The limited anatomical resolution of SPECT imaging in rodents with small lungs hampers precise evaluation of the distribution of ^99m^Tc-PulmoBind and the detection of heterogeneity of distribution. In a larger animals (dog) and in humans, we showed that ^99m^Tc-PulmoBind lung distribution follows gravitational and non-gravitational gradients in normal conditions that are modified by pathology [18]. Furthermore, in humans with group I PH, we derived a “heterogeneity perfusion index” and showed its utility in quantifying abnormal distribution of ^99m^Tc-PulmoBind in these subjects [10]. Better analytical quantification is therefore expected in human subjects.

## Conclusion

SPECT imaging with the molecular endothelial tracer ^99m^Tc-PulmoBind detects PVD and its severity in bleomycin-induced lung fibrosis. Further development of this tracer for imaging of PVD associated with chronic lung disorders such as IPF is supported.

## Supplementary Information

**Additional file 1: Fig. S1.** Reduced ^99m^Tc-PulmoBind uptake is associated with the severity of pulmonary vascular disease. Simple linear regression of lung ^99m^Tc-PulmoBind with the severity of pulmonary hypertension (PAAT) and right ventricular dysfunction (TAPSE) in the right and left lung. PAAT: Pulmonary artery acceleration time, TAPSE: Tricuspid annulus plane systolic excursion.

## Data Availability

The datasets used and/or analyzed during the current study are available from the corresponding author on reasonable request.

## Abbreviations

AM: Adrenomedullin
αSMA: Alpha-smooth muscle actin
CLR: Calcitonin-like receptor
IPF: Idiopathic pulmonary fibrosis
MAA: Macroaggregates of albumin
PAAT: Pulmonary artery acceleration time
PAP: Pulmonary artery pressure
PH: Pulmonary hypertension
PVD: Pulmonary vascular disease
RAMP2: Receptor activity modifying protein 2
RV: Right ventricle
RVAWd: Right ventricle anterior wall thickness at end-diastole
RVDd: Right ventricle dimension at end-diastole
SM22a: Smooth muscle protein 22-alpha
SPECT: Single photon emission computed tomography
TAPSE: Tricuspid annulus plane systolic excursion
Tc: Technetium
