# Supplementary figures and images for: SPECT imaging of pulmonary vascular disease in bleomycin-induced lung fibrosis using a vascular endothelium tracer
Source: Respir Res. 2021 Sep 4;22:240. doi: 10.1186/s12931-021-01836-3 (PMC8418741; doi:10.1186/s12931-021-01836-3)

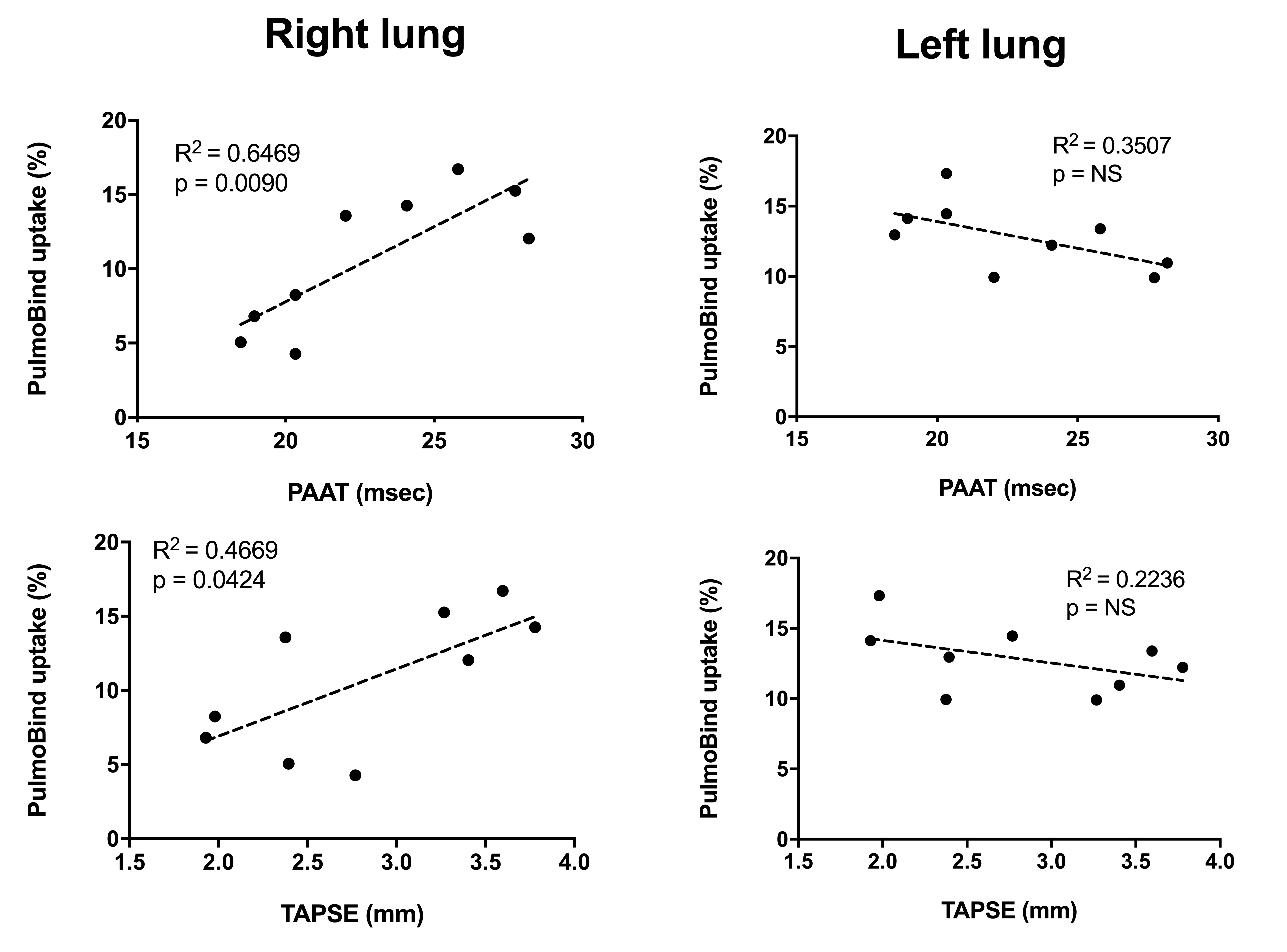

Supplement: Supplementary file 1 — Additional file 1: Fig. S1. Reduced 99mTc-PulmoBind uptake is associated with the severity of pulmonary vascular disease. Simple linear regression of lung 99mTc-PulmoBind with the severity of pulmonary hypertension (PAAT) and right ventricular dysfunction (TAPSE) in the right and left lung. PAAT: Pulmonary artery acceleration time, TAPSE: Tricuspid annulus plane systolic excursion. [file 12931_2021_1836_MOESM1_ESM.tiff]
